# Risk of anaphylaxis in opioid dependent persons: effects of heroin versus substitution substance

**DOI:** 10.1186/1747-597X-9-12

**Published:** 2014-02-27

**Authors:** Ute Maurer, Carola Kager, Christina Fellinger, Dagmara Loader, Augustin Pollesböck, Bernhard Spitzer, Reinhart Jarisch

**Affiliations:** 1 Department: Health, Biomedical Science, University of Applied Sciences Wiener Neustadt, Johannes Gutenberg-Strasse 3, 2700 Wiener Neustadt, Austria; 2 Floridsdorfer Allergy Centre (FAZ), Franz Jonasplatz 8, 1210 Vienna, Austria; 3 Landesklinikum Mauer, Withdrawal Centre, Hausmeninger Straße 221, 3362 Mauer/Amstetten, Austria

**Keywords:** Drug Addiction, Anaphylaxis, Histamine, Opioid

## Abstract

**Background:**

Across Europe, illicit drug-related mortality has not declined despite ever increasing prevention measures. The cause of these deaths has traditionally been associated with overdose. Previous findings have revealed the appearance of non-lethal opioid concentrations, leading us to investigate a further cause of death. The symptoms of heroin intoxication with asphyxia and/or cardiovascular involvement resemble anaphylaxis, and therefore it has been speculated that such deaths might be caused by an allergic reaction. The study´s aims were to investigate levels of allergic mediators in long-term injecting drug users (IDU) compared to healthy controls and to determine if oral opioid substitution therapy (OST) resulted in similar allergic symptoms to those reported by IDU after intravenous (IV) heroin use.

**Methods:**

We quantified the concentrations of histamine, diamine oxidase (DAO), tryptase and lipoprotein-associated phospholipase A_2_ (LpPLA_2_) at baseline and 1 h after administration of Substitol®retard (482 ± 220 mg) in 56 patients at a withdrawal centre (Austria) and compared them with healthy controls (n = 103). Questionnaires and face-to-face interviews were used to assess allergic symptoms and side effects in IDU. Descriptive statistical analyses of quantitative data were performed by using SPSS.

**Results:**

Baseline histamine, tryptase and LpPLA_2_ were significantly elevated in IDU compared to the healthy control group, while DAO decreased. Blood levels showed no significant change after oral substitution uptake. Self-reported allergic symptoms and side effects after IV heroin use were reported in 55 cases (98.2%), minimal symptoms were documented after OST (12.5%, 7/56).

**Conclusions:**

This study revealed that baseline histamine concentrations were elevated in chronic IDU, although only relatively small changes in tryptase plasma levels occurred. After IV heroin application the reported allergic symptoms were mostly mild and did not lead to clinically relevant side effects. The substitution substance was clearly better tolerated than IV administered heroin. Elevated levels of allergic mediators such as histamine in IDUs may place them at greater risk of severe or fatal anaphylaxis when exposed to heroin; however, this requires further investigation.

## Background

Drug-related mortality is often marked by a lack of demonstrable cause of death. Death due to overdose may be misleading in many cases [1-3]. Several studies have shown a wide range of morphine concentrations, usually non-lethal, in post-mortem samples with coincidental poly drug-use [1,4-6], leading us to investigate a further cause of death. The symptoms of heroin intoxication with asphyxia and/or cardiovascular involvement resemble anaphylaxis, and therefore it has been speculated that such deaths might be caused by an allergic reaction.

Anaphylaxis is a severe, potentially life-threatening allergic reaction affecting multiple organ systems - including the skin, respiratory tract, gastrointestinal tract and central nervous system [7-10]. Common triggers of anaphylaxis include certain foods, insect venom and some medications [10,11]. The essential common factor in anaphylaxis, whether by immunological or non- immunological mechanisms, is the activation of mast cells and circulating basophils, with the release of preformed vasoactive mediators (such as histamine, mast cell tryptase [MCT] and chymase), newly synthesized cytokines as well as lipid-derived mediators [12-16]. These mediators act on target cells and can induce vasodilation, increased permeability, hypotension, bronchospasm and as a result, shock. The symptomatology is variable; there is no obligatory involvement of all organ systems [17,18]. Cutaneous manifestations (such as pruritus, flushing, urticaria and angioedema) are by far the most common. But reactions can progress within minutes to respiratory and/or cardiovascular arrest with lethal ending.

The diagnosis of anaphylaxis is based primarily on the clinical picture and history which can be corroborated by serologic laboratory tests. Currently, there is no in vitro test for diagnosing anaphylaxis but the serial measurement of immune mediators, such as histamine, MCT as well as other products of mastocytes or basophils, may be useful for confirming diagnosis and potentially identifying risk [19]. Diagnostic criteria for anaphylaxis were published by a multidisciplinary group of experts [18] who agreed on the lack of information about reliable biomarkers to confirm the clinical impression. Sometimes it is not feasible to obtain samples within the optimum time frame [20]. Moreover, even with early collection of samples, histamine and tryptase may be within normal levels [20]. Therefore they are described as nonspecific markers for anaphylaxis which can be increased in other disease as well [21].

Many opioids are known to stimulate mast cell and basophil degranulation non-specifically [22,23]. There are differences in their capacity to cause histamine release along with other mediators as well in variable opioid-dependent effects [24].

In the present study, we investigated allergic symptoms and side effects in IDU after IV heroin injection (self-reported) and after opioid substitution treatment (OST) (observed) within 180 minutes. To determine if long-term IV heroin use result in altered biomarker levels, we analyzed allergic mediators in opioid dependent persons (baseline and after OST) and compared with healthy controls.

## Methods

### Study design

The objectives were addressed by performing quantitative laboratory analysis (immunoassays) and qualitative data analysis (questionnaires, face-to-face interviews).

Blood samples were obtained from opioid dependent persons at a withdrawal centre at Amstetten/Mauer (Austria) who had been undergoing an OST program for 2 weeks. Samples from IDU were taken prior (baseline) and 1 hour after substitution treatment. The first blood sampling was conducted 10 minutes before morning administration. Subsequent sampling proceeded 1 hour after oral intake of substitution substance. Blood analysis served in the determination of baseline allergic biomarkers, such as histamine, diamine oxidase (DAO), tryptase and lipoprotein associated phospholipase A_2_ (LpPLA_2_). Serum and plasma samples were collected into plain and EDTA vacutainers, using VACUETTE® products manufactured by Greiner Bio-One. The tubes were transported in dry ice and centrifuged within 30 minutes after sampling. After centrifugation at 3000U/min for 10 minutes, samples were immediately stored at -20°C until analysis. Laboratory analyses were performed at the Floridsdorfer Allergy Centre (FAZ), Vienna (Austria).

Supplementary, the clinical picture of long-term IDU before and after substitution was documented. Therefore face-to-face interviews and questionnaire procedures were used. A structured datasheet was used to record the following factors: demographics, characteristics of allergen/trigger exposure (kind of opioid, daily dosage, mean duration of regular abuse, etc.), pre-existent allergic conditions and/or mastocytosis as well as self-reported allergic reactions and side-effects shown during former IV drug consumption. After substitution, a structured datasheet was filled in to record treatments, side effects and individual reaction features after 15, 30, 60, and 180 minutes.

### Study population

All participants gave their written informed consent. Approval was also obtained from relevant federal authorities and the ethics committee.

At the withdrawal centre in Amstetten/Mauer, 78 drug addicts (58 male/ 20 female) were tested from a patient population. The key inclusion criteria for OST were a main diagnosis of opiate dependence according to ICD-10 [25] as well as suitable venous conditions for blood sampling. Patients’ age, sex, or ethnic background were not considered as recruitment criteria for this study. The presence of underlying allergic disease was reported in 22 of 78 cases, such as medication (n = 11), grass pollen (n = 8), food (n = 3) and insect sting (n = 2) allergies. Further clinical risk factors for anaphylaxis, comprising comorbidities such as asthma, cardiovascular disease or mastocytosis, were not recognized. In the final analysis, 56 IDU without underlying allergic disease, were included in the study population.

Most subjects had a significant history of regular opioids abuse, ranging from 2 to 25 years. The mean duration of regular heroin abuse was 7 ± 5.3 years. All participants at the withdrawal centre in Amstetten/Mauer were treated with Substitol®retard. In Austria, Substitol found its role in OST programs to treat heroin addiction as a controlled, slow releasing oral medicine. It is a semi-synthetic, highly potent and long acting opioid analgesic [26]. Single substance doses varied from 120 to 1000 mg (482 ± 220 mg), depending on type of substance, duration of intake and daily dosage.

For the comparison group volunteers (n = 103) were recruited during the same period as the drug addicts between October 2011 and December 2012. 38 male and 65 female persons were included, ages varied from 18 to 61 years (age 31.4 ± 11.8). Subjects with known allergic diseases were excluded.

## Laboratory tests

### Histamine

Histamine, a biogenic amine, is the most important inflammatory mediator released by degranulation of mast cells and basophils during an allergic reaction [27,28]. Many opioids are known to be potent histamine releasers [29,30]. Therefore it is of high interest to evaluate baseline histamine concentrations in chronic IDUs. Histamine levels were determined by radioimmunoassay (Immunotec, France) [31]. In brief, blood was collected in a chilled tube containing EDTA and immediately cooled on ice. Samples were centrifuged for 10 minutes at 900 g/4°C. Acylation buffer and calibrator were then added and immediately vortexed. Results were obtained from the standard curve by interpolation. Normal histamine levels in living subjects are <0.3 ng/ml [32].

### Diamine oxidase (DAO)

DAO is a major histamine-degrading enzyme which is found in various tissues, but is primarily active in the intestinal mucosa [33]. DAO activity in serum samples of healthy individuals normally ranged from 10 to 30U/ml. Lower DAO activity was described as a potential indicator for intestinal mucosa damage in inflammatory and neoplastic disease [34] as well as in cases of fatal anaphylaxis [35]. The activity of DAO was determined by quantitating the reaction product (Sciotec Diagnostics Tulln, Austria) [36]. Radiolabelled putrescine-dihydrochloride was used as a substrate. The result Δ^1^pyrroline, containing the radiolabel, was extracted selectively from the matrix by a liquid extraction step. A non-toxic, chlorine-free solvent with high capacity was used for extraction. Finally scintillation fluid was added to the organic phase containing the radiolabelled Δ^1^-pyrroline and radioactivity was determined in a beta-counter. The signal was directly proportional to the activity of DAO in the sample. Results < 11U/ml are judged to be reduced.

### Tryptase

Mast cell tryptase (MCT) is a tetrameric neutral serine protease which is nearly exclusive to mast cells [16,37]. It is more stable, has a longer half-life than histamine and can be detected from a few minutes up to several hours after mast cell degranulation [20,38]. Anti-Tryptase, covalently bound to a solid phase reacted with the tryptase in the serum (Thermo Fisher Scientific, Austria) [39]. After a washing procedure, enzyme labelled antibodies against tryptase was added to form a complex. After incubation, unbound enzyme anti-tryptase was washed away and the bound complex incubated with a developer. After stopping the reaction, the fluorescense of the eluate was measured. The fluorescence was directly proportional to the tryptase concentration of the serum. Normal levels were <11.4 μg/l.

### Lipoprotein-associated Phospholipase A_2_

Lipoprotein-associated phospholipase A_2_ (LpPLA_2_), a Ca_2_+ independent phospholipase A_2_, was identified in human plasma and found to be responsible for hydrolysis and inactivation of platelet-activating factor (PAF) and certain oxidized phospholipids [40]. Although the role of LpPLA_2_ as a pro- or anti-atherosclerotic enzyme is frequently debated, several studies have shown it to be an independent marker of cardiovascular disease [41,42]. This method (diaDexus, South San Francisco) [43] utilizes monoclonal anti-LpPLA_2_ antibodies directed against LpPLA_2_ for solid phase immobilization on the micro well plate. Serum samples were added to the plate and after incubation, second monoclonal anti-LpPLA_2_ antibody labelled with the enzyme horseradish peroxidase, were added and then incubated with the immobilized antigen. The added substrate, tetramethylbenzidine, followed the immunological reaction. The absorbance of the enzymatic turnover of the substrate was determined by spectrophotometry at 450 nm. It was directly proportional to the present concentration of LpPLA_2_.

### Statistical analysis

Descriptive statistical analyses of quantitative data were performed by using SPSS, version 20. Comparisons between groups were made by using the Mann-Withney Test. Data was expressed as mean ± SD. The level of significance was set at 5%.

## Results

**Figure 1:**
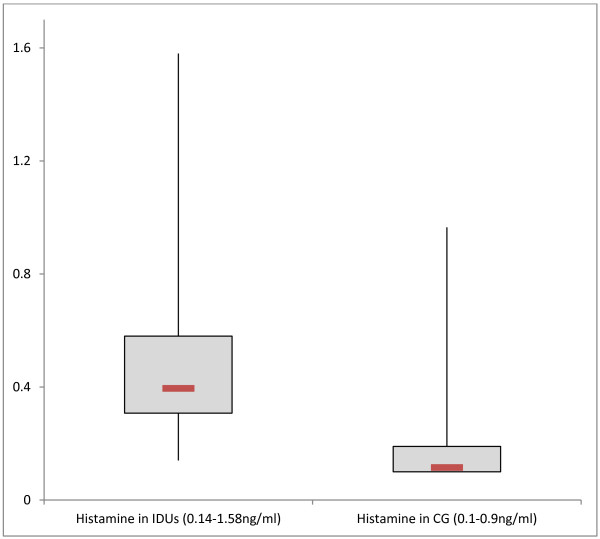
Baseline histamine levels (ng/ml) measured in injecting drug users (IDUs) and in comparison group (CG), (p < 0.001, Mann-Whitney Test).

**Figure 2:**
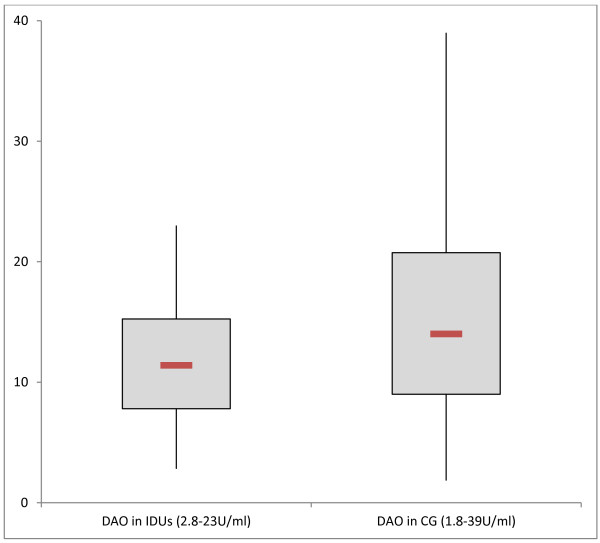
Baseline DAO levels (U/ml) measured in injecting drug users (IDUs) and in comparison group (CG), (p < 0.05, Mann-Whitney Test).

**Figure 3:**
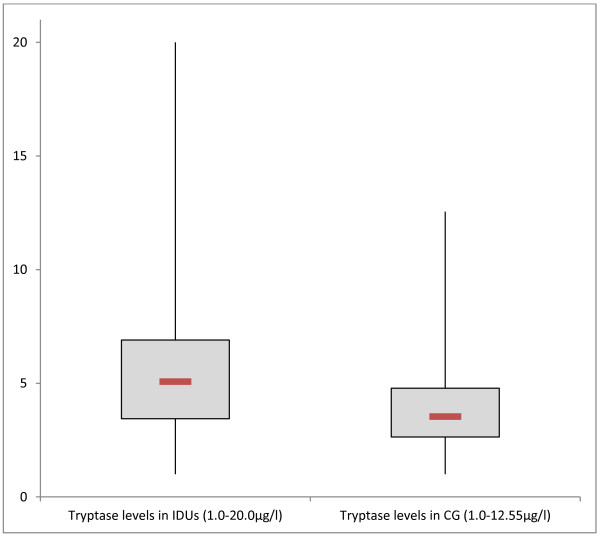
Baseline tryptase levels (μg/l) measured in injecting drug users (IDUs) and in comparison group (CG), (p < 0.001, Mann-Whitney Test).

**Figure 4:**
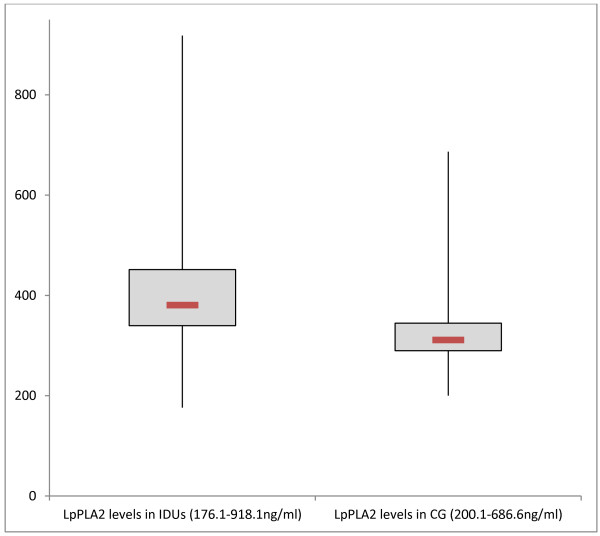
**Baseline LpPLA**_**2 **_**levels (ng/ml) measured in injecting drug users (IDUs) and in comparison group (CG), (p < 0.001, Mann-Whitney Test).**

At baseline, histamine levels were elevated in IDU (0.50 ± 0.30 ng/ml, 95% CI 0.42-0.58 ng/ml) compared to healthy controls (0.18 ± 0.15 ng/ml, 95% CI 0.15-0.21 ng/ml) (p < 0.001, Mann-Whitney Test), shown in Figure 1. We found increased baseline histamine levels (>0.3 ng/ml) in 45 of 56 IDUs. Baseline DAO concentrations were decreased in opioid dependent persons (11.8 ± 4.9U/ml, 95% CI 10.43-13.21U/ml), compared to controls (15.7 ± 7.7U/ml, 95% CI 14.19-17.16U/ml) (p < 0.05, Mann-Whitney Test; see Figure 2). In chronic drug abusers, tryptase values varied between 1 and 20 μg/l (6.0 ± 4.3 μg/l, 95% CI 4.89-7.18 μg/l; Figure 3). In the healthy controls tryptase values varied between 1 and 12.5 μg/l (3.9 ± 1.9 μg/l, 95% CI 3.58-4.34 μg/l; p < 0.001, Mann-Whitney Test). We found high LpPLA_2_ levels in IDUs (411.9 ± 125 ng/ml, 95% CI 377.27-446.58 ng/ml) versus 333.3 ± 85.9 ng/ml (95% CI 316.76- 349.80 ng/ml) in healthy controls (p < 0.001, Mann-Whitney Test; Figure 4).

Blood levels of all markers showed no significant change after OST.

After IV heroin, 55 of 56 IDU (98.2%) self-reported allergic symptoms and side effects. The most common manifestations were cutaneous symptoms which occurred within the first 15 minutes, including flushing (69.6%), urticaria (57.1%), pruritus (55.4%) and itching of the palms and feet (46.4%). Respiratory obstruction (dyspnea in 42.9%) and arrhythmias (51.8%) were also reported after heroin use.

In contrast, mild symptoms were documented in 7 of 56 cases (12.5%) after OST within 180 minutes. All of them showed cutaneous symptoms, including itching of the palms and feet, pruritus and flushing as well as headache and tiredness.

## Discussion

Baseline histamine levels were almost 3-fold higher in chronic IV opioid abusers than in healthy controls. Many opiates are known to induce histamine release by mast cells and basophils causing various effects like vasodilatation, bronchospasm and pruritus [44,45]. However, several studies have shown that opioids differ in their capacity to cause histamine release and vary in opioid-dependent effects [22,30,46]. Divergent study results can be explained with the variable dosage of opioids, its mode and rate of administration, the distribution of histamine receptors in different tissues, the effects of concomitant medications and, the heterogeneity of patient responses to histamine [24,47,48]. Histamine is a non-specific marker for allergic reactions with a very short half-life [20]. In the present study it is unlikely that IV heroin is directly responsible for the observed histamine concentrations. It is revealing that elevated histamine may be a consequence of chronic immune activation due to regular heroin use and/ or co-morbidities. In addition to heavy drug use associated with non-immunological symptoms and side effects, immunoglobulin E (IgE) antibody-mediated allergic reactions to opioids which might be rare have to be considered. In such cases skin tests with the suspected drug, drug-specific IgE antibody assays, and flow cytometric activation of basophils with simultaneous analysis of CD63 appearance may help diagnosis [49].

The immune response is further regulated by the balance of both histamine and DAO. Reduced DAO levels were found in IDU (29/56) which might signify low histamine-degrading capacity, allowing a prolonged presence of this vasoactive mediator. Therefore, a reason for elevated histamine in IDU may be competitive inhibition of histamine degradation of DAO by other biogenic amines, alcohol or drugs. Hence, low DAO levels do not seem to be specifically associated with anaphylactic reactions.

Although the mean plasma tryptase levels at baseline were significantly different in IDU versus the comparison group, only 4 of 56 cases tryptase values were above 11.4 μg/l showing marginal elevations (20, 18.8, 17.4 and 17.3 μg/l). A relationship between fatal anaphylaxis and elevated tryptase levels was shown in previous studies [35,50]. Edston et al. [51] found elevated tryptase levels in one third of patients who died after heroin intoxication; however the heroin level was not high enough to explain the fatalities. Rook et al. [52] reported increased tryptase concentrations after IV heroin injection in chronic opioid users, but not after heroin inhalation. This may be explained by the type of entry itself which may affect the type of response to the injected substance.

In the present study LpPLA_2_ concentrations showed an unexpectedly wide range of variation in both groups. We observed an up-regulation of LpPLA_2_ in IDU. It suggests that increased expression of this enzyme may be a physiological response to inflammatory stimuli. In this context, we have to mention that activated basophils can induce systemic anaphylaxis through the release of the potent platelet-activating factor (PAF) upon stimulation with immune complexes, even though they present less than 1% of leukocytes in the body [53,54]. Therefore LpPLA_2_ is attributed to play a pivotal role in anaphylaxis as well as in arteriosclerosis [55].

Our findings clearly indicate that long-term IV heroin application and oral uptake of Substitol®retard gave rise to markedly different drug effects. Self-reported allergic symptoms and side-effects after regular IV heroin application were documented in 55 of 56 cases. Most commonly cutaneous manifestations (flushing, urticaria, pruritus) were reported. But in context of the clinical criteria for diagnosing anaphylaxis, skin symptoms alone are not indicating an anaphylactic event. Underlying disease as well as the interference of alcohol or other drugs taken must be examined to be better understood in this context. These findings may be explained by the physiological effects of the injected heroin itself. A report by Haemmig et al. [56], to evaluate the effects of high doses of injected opiates as prescribed in maintenance of IV drug users, showed that in 16 of 39 cases, the study has to be discontinued owing to severe morphine-induced histamine reactions, such as pruritus, urticaria, nausea and flushing. Further studies by Grossmann et al. [57] and Schug et al. [58] described comparable symptoms related to histamine, like itching around the injection site, peripheral vasodilatation and asthmatic attacks after the use of opioids. Though, cases with respiratory involvement in addition with skin features may indicate a moderately severe anaphylactic event.

In contrast minimal symptoms, mostly unspecific ones, were seen after oral substitution medication in 7 of 56 cases. Oral substitution substance was clearly better tolerated than IV administered heroin. These results may indicate a well personalized substitution therapy administered by the medical staff of the withdrawal clinic.

The study is limited by small sample size and lack of adequate information on the general health condition of IDU. Obviously, long-term IV opioid dependent persons suffer poorer health due to the complications of drug use characterized by cardiovascular diseases, pulmonary diseases, renal complications, dental problems and gynaecological issues in females. Notwithstanding, hepatitis B, hepatitis C and human immunodeficiency virus (HIV) are the predominant infectious diseases in drug addicts which lead to a weak immune system in this population [59,60].

## Conclusions

This effort to open the ‘black box’ of anaphylaxis is critical to our efforts to understand this condition among IV heroin abusers. This study revealed that baseline histamine concentrations were elevated in chronic IDU, although only relatively small changes in tryptase plasma levels occurred. After IV heroin application the reported allergic symptoms were mostly mild and did not lead to clinically relevant side effects. The substitution substance was clearly better tolerated than IV administered heroin. Elevated levels of allergic mediators such as histamine in IDUs may place them at greater risk of severe or fatal anaphylaxis when exposed to heroin; however, this requires further investigation.

## Abbreviations

IDU: Injecting drug users; OST: Opioid substitution therapy; DAO: Diamine oxidase; LpPLA2: Lipoprotein-associated phospholipase A_2_; MCT: Mast cell tryptase; IV: intravenous.

## Competing interests

The authors declare that they have no competing interests. Funding was done by the Floridsdorfer Allergy Centre (FAZ), Vienna.

## Authors' contributions

UM was involved in the research design, carried out the data collection, and drafted the manuscript. CK, CF, DL and AP contributed substantive content and crucial revisions to the manuscript. RJ and BS were involved in the clinical arrangement, the data collection and drafted the manuscript. All authors have reviewed and approved the manuscript submitted.

## References

[B1] Risser D Honigschnabl S Stichenwirth M Pfudl S Sebald D Kaff A Bauer G Mortality of opiate users in Vienna, Austria Drug Alcohol Depend 2001 64 3 251 256 10.1016/S0376-8716(01)00131-4 11672939

[B2] Risser D Schneider B Drug-related deaths between 1985 and 1992 examined at the Institute of Forensic Medicine in Vienna, Austria Addiction 1994 89 7 851 857 10.1111/j.1360-0443.1994.tb00988.x 8081183

[B3] Darke S Zador D Fatal heroin 'overdose': a review Addiction 1996 91 12 1765 1772 10.1111/j.1360-0443.1996.tb03800.x 8997759

[B4] Staub C Jeanmonod R Fryc O Morphine in postmortem blood: its importance for the diagnosis of deaths associated with opiate addiction Int J Legal Med 1990 104 1 39 42 10.1007/BF01816482 11453091

[B5] Mathers BM Degenhardt L Bucello C Lemon J Wiessing L Hickman M Mortality among people who inject drugs: a systematic review and meta-analysis Bull World Health Organ 2013 91 2 102 123 10.2471/BLT.12.108282 23554523 PMC3605003

[B6] Polettini A Poloni V Groppi A Stramesi C Vignali C Politi L Montagna M The role of cocaine in heroin-related deaths. Hypothesis on the interaction between heroin and cocaine Forensic Sci Int 2005 153 1 23 28 10.1016/j.forsciint.2005.04.017 16039419

[B7] Simons FE 9. Anaphylaxis J Allergy Clin Immunol 2008 121 2 Supp S402 S407 quiz S420 18241691 10.1016/j.jaci.2007.08.061

[B8] Simons FE Anaphylaxis J Allergy Clin Immunol 2010 125 2 Suppl 2 S161 S181 20176258 10.1016/j.jaci.2009.12.981

[B9] Simons FE World Allergy Organization survey on global availability of essentials for the assessment and management of anaphylaxis by allergy-immunology specialists in health care settings Ann Allergy Asthma Immunol 2010 104 5 405 412 10.1016/j.anai.2010.01.023 20486330

[B10] Ben-Shoshan M Clarke AE Anaphylaxis: past, present and future Allergy 2011 66 1 1 14 10.1111/j.1398-9995.2010.02422.x 20560905

[B11] Pumphrey R Anaphylaxis: can we tell who is at risk of a fatal reaction? Curr Opin Allergy Clin Immunol 2004 4 4 285 290 10.1097/01.all.0000136762.89313.0b 15238794

[B12] Tsai MG S. G Castells MC Mast cells: effector cells of anaphylaxis Anaphylaxis and Hypersensitivity Reactions 2011 New York: Humana Press 47 68

[B13] Lee JK Vadas P Anaphylaxis: mechanisms and management Clin Exp Allergy 2011 41 7 923 938 10.1111/j.1365-2222.2011.03779.x 21668816

[B14] Stone SF Cotterell C Isbister GK Holdgate A Brown SG Elevated serum cytokines during human anaphylaxis: identification of potential mediators of acute allergic reactions J Allergy Clin Immunol 2009 124 4 786 792 e784 10.1016/j.jaci.2009.07.055 19767073

[B15] Stone KD Prussin C Metcalfe DD IgE, mast cells, basophils, and eosinophils J Allergy Clin Immunol 2010 125 2 Suppl 2 S73 S80 20176269 10.1016/j.jaci.2009.11.017 PMC2847274

[B16] Schwartz LB Effector cells of anaphylaxis: mast cells and basophils Novartis Found Symp 2004 257 65 74 discussion 74-69, 98-100, 276-185 15025392

[B17] Simons FE Sampson HA Anaphylaxis epidemic: fact or fiction? J Allergy Clin Immunol 2008 122 6 1166 1168 10.1016/j.jaci.2008.10.019 19084110

[B18] Sampson HA Munoz-Furlong A Campbell RL Adkinson NF Jr Bock SA Branum A Brown SG Camargo CA Jr Cydulka R Galli SJ Gidudu J Gruchalla RS Harlor AD Jr Hepner DL Lewis LM Lieberman PL Metcalfe DD O'Connor R Muraro A Rudman A Schmitt C Scherrer D Simons FE Thomas S Wood JP Decker WW Second symposium on the definition and management of anaphylaxis: summary report–Second National Institute of Allergy and Infectious Disease/Food Allergy and Anaphylaxis Network symposium J Allergy Clin Immunol 2006 117 2 391 397 10.1016/j.jaci.2005.12.1303 16461139

[B19] Sanz ML Gamboa PM Garcia-Figueroa BE Ferrer M Ring J In vitro Diagnosis of Anaphylaxis Anaphylaxis Chemical Immunology and Allergy, Volume 95 2010 Basel: Kager 125 140 10.1159/000315947 20519886

[B20] Schwartz LB Yunginger JW Miller J Bokhari R Dull D Time course of appearance and disappearance of human mast cell tryptase in the circulation after anaphylaxis J Clin Invest 1989 83 5 1551 1555 10.1172/JCI114051 2468689 PMC303860

[B21] Schwartz LB Diagnostic value of tryptase in anaphylaxis and mastocytosis Immunol Allergy Clin North Am 2006 26 3 451 463 10.1016/j.iac.2006.05.010 16931288

[B22] Baldo BA Pham NH Histamine-releasing and allergenic properties of opioid analgesic drugs: resolving the two Anaesth Intensive Care 2012 40 2 216 235 22417016 10.1177/0310057X1204000204

[B23] Veien M Szlam F Holden JT Yamaguchi K Denson DD Levy JH Mechanisms of nonimmunological histamine and tryptase release from human cutaneous mast cells Anesthesiology 2000 92 4 1074 1081 10.1097/00000542-200004000-00026 10754628

[B24] Stellato C Cirillo R de Paulis A Casolaro V Patella V Mastronardi P Mazzarella B Marone G Human basophil/mast cell releasability. IX. Heterogeneity of the effects of opioids on mediator release Anesthesiology 1992 77 5 932 940 10.1097/00000542-199211000-00016 1280014

[B25] Organization WH ICD-10, the ICD-10 Classification of Mental and Behavioural Disorders: Diagnostic Criteria for Research 1993 World Health Organization

[B26] Union E EU Drugs Strategy (2013-2020) 2012

[B27] Dale HH Laidlaw PP The physiological action of beta-iminazolylethylamine J Physiol 1910 41 5 318 344 16993030 10.1113/jphysiol.1910.sp001406 PMC1512903

[B28] Parsons ME Ganellin CR Histamine and its receptors Br J Pharmacol 2006 147 Suppl 1 S127 S135 16402096 10.1038/sj.bjp.0706440 PMC1760721

[B29] Di Bello MG Masini E Ioannides C Ndisang JF Raspanti S Bani Sacchi T Mannaioni PF Histamine release from rat mast cells induced by the metabolic activation of drugs of abuse into free radicals Inflamm Res 1998 47 3 122 130 10.1007/s000110050299 9562337

[B30] Hermens JM Ebertz JM Hanifin JM Hirshman CA Comparison of histamine release in human skin mast cells induced by morphine, fentanyl, and oxymorphone Anesthesiology 1985 62 2 124 129 10.1097/00000542-198502000-00005 2578752

[B31] Immunotech BCC RIA Histamine, Immunradiometrischer Assay für die quantitative in vitro Bestimmung von Histamin in biologischen Proben Immunotech SAS 1 18

[B32] Jarisch R Histamin-Intoleranz, Histamin und Seekrankheit 2013 Stuttgart: Thieme Verlag

[B33] Luk GD Bayless TM Baylin SB Diamine oxidase (histaminase): a circulating marker for rat intestinal mucosal maturation and integrity J Clin Invest 1980 66 1 66 70 10.1172/JCI109836 6772669 PMC371506

[B34] Raithel M Ulrich P Hochberger J Hahn EG Measurement of gut diamine oxidase activity. Diamine oxidase as a new biologic marker of colorectal proliferation? Ann N Y Acad Sci 1998 859 262 266 10.1111/j.1749-6632.1998.tb11142.x 9928401

[B35] Mayer DE Krauskopf A Hemmer W Moritz K Jarisch R Reiter C Usefulness of post mortem determination of serum tryptase, histamine and diamine oxidase in the diagnosis of fatal anaphylaxis Forensic Sci Int 2011 212 1–3 96 101 21664082 10.1016/j.forsciint.2011.05.020

[B36] Diaminoxidase (DAO), Radioextraktionsassay (REA) zur quantitativen Bestimmung der DAO-Aktivität Serum und Plasma [http://www.sciotec.at/en/products/diagnostics/]

[B37] Schwartz LB Metcalfe DD Miller JS Earl H Sullivan T Tryptase levels as an indicator of mast-cell activation in systemic anaphylaxis and mastocytosis N Engl J Med 1987 316 26 1622 1626 10.1056/NEJM198706253162603 3295549

[B38] Payne V Kam PC Mast cell tryptase: a review of its physiology and clinical significance Anaesthesia 2004 59 7 695 703 10.1111/j.1365-2044.2004.03757.x 15200544

[B39] Test Principle ImmunoCAP Tryptase [http://www.phadia.com/da/Products/Allergy-testing-products/ImmunoCAP-Assays/ImmunoCAP-Tryptase/]

[B40] Dada N Kim NW Wolfert RL Lp-PLA2: an emerging biomarker of coronary heart disease Expert Rev Mol Diagn 2002 2 1 17 22 10.1586/14737159.2.1.17 11963798

[B41] Carlquist JF Muhlestein JB Anderson JL Lipoprotein-associated phospholipase A2: a new biomarker for cardiovascular risk assessment and potential therapeutic target Expert Rev Mol Diagn 2007 7 5 511 517 10.1586/14737159.7.5.511 17892360

[B42] Zalewski A Nelson JJ Hegg L Macphee C Lp-PLA2: a new kid on the block Clin Chem 2006 52 9 1645 1650 10.1373/clinchem.2006.070672 16873290

[B43] The PLAC Test ELISA Kit [http://www.plactest.com/international/package-insert-plac-test-elisa-kit]

[B44] Hirshman CA Downes H Butler S Relevance of plasma histamine levels to hypotension Anesthesiology 1982 57 5 424 426 10.1097/00000542-198211000-00018 7137626

[B45] Fahmy NR Sunder N Soter NA Role of histamine in the hemodynamic and plasma catecholamine responses to morphine Clin Pharmacol Ther 1983 33 5 615 620 10.1038/clpt.1983.83 6839633

[B46] Barke KE Hough LB Opiates, mast cells and histamine release Life Sci 1993 53 18 1391 1399 10.1016/0024-3205(93)90581-M 7694026

[B47] Marone G Stellato C Mastronardi P Mazzarella B Mechanisms of activation of human mast cells and basophils by general anesthetic drugs Annales francaises d'anesthesie et de reanimation 1993 12 2 116 125 10.1016/S0750-7658(05)81020-2 7690200

[B48] Levy JH Brister NW Shearin A Ziegler J Hug CC Jr Adelson DM Walker BF Wheal and flare responses to opioids in humans Anesthesiology 1989 70 5 756 760 10.1097/00000542-198905000-00008 2470272

[B49] Leysen J De Witte L Sabato V Faber M Hagendorens M Bridts C De Clerck L Ebo D IgE-mediated allergy to pholcodine and cross-reactivity to neuromuscular blocking agents: Lessons from flow cytometry Cytometry B Clin Cytom 2013 84 2 65 70 23355309 10.1002/cyto.b.21074

[B50] Edston E van Hage-Hamsten M beta-Tryptase measurements post-mortem in anaphylactic deaths and in controls Forensic Sci Int 1998 93 2-3 135 142 10.1016/S0379-0738(98)00040-1 9717264

[B51] Edston E van Hage-Hamsten M Anaphylactoid shock–a common cause of death in heroin addicts? Allergy 1997 52 9 950 954 10.1111/j.1398-9995.1997.tb01256.x 9298181

[B52] Rook EJ Hillebrand MJ Rosing H van Ree JM Beijnen JH The quantitative analysis of heroin, methadone and their metabolites and the simultaneous detection of cocaine, acetylcodeine and their metabolites in human plasma by high-performance liquid chromatography coupled with tandem mass spectrometry J Chromatogr B Analyt Technol Biomed Life Sci 2005 824 1–2 213 221 16103023 10.1016/j.jchromb.2005.05.048

[B53] Karasuyama H Mukai K Tsujimura Y Obata K Newly discovered roles for basophils: a neglected minority gains new respect Nat Rev Immunol 2009 9 1 9 13 10.1038/nri2458 19039320

[B54] Mukai K Obata K Tsujimura Y Karasuyama H [The roles for basophils in allergy] Nihon Rinsho 2009 67 11 2095 2099 19899522

[B55] Khuseyinova N Imhof A Rothenbacher D Trischler G Kuelb S Scharnagl H Maerz W Brenner H Koenig W Association between Lp-PLA2 and coronary artery disease: focus on its relationship with lipoproteins and markers of inflammation and hemostasis Atherosclerosis 2005 182 1 181 188 10.1016/j.atherosclerosis.2004.10.046 16115490

[B56] Haemmig RB Tschacher W Effects of high-dose heroin versus morphine in intravenous drug users: a randomised double-blind crossover study J Psychoactive Drugs 2001 33 2 105 110 10.1080/02791072.2001.10400475 11476257

[B57] Grossmann M Abiose A Tangphao O Blaschke TF Hoffman BB Morphine-induced venodilation in humans Clin Pharmacol Ther 1996 60 5 554 560 10.1016/S0009-9236(96)90151-4 8941028

[B58] Schug SA Zech D Grond S Adverse effects of systemic opioid analgesics Drug Saf 1992 7 3 200 213 10.2165/00002018-199207030-00005 1354445

[B59] Nelson PK Mathers BM Cowie B Hagan H Des Jarlais D Horyniak D Degenhardt L Global epidemiology of hepatitis B and hepatitis C in people who inject drugs: results of systematic reviews Lancet 2011 378 9791 571 583 10.1016/S0140-6736(11)61097-0 21802134 PMC3285467

[B60] Eskild A Magnus P Samuelsen SO Sohlberg C Kittelsen P Differences in mortality rates and causes of death between HIV positive and HIV negative intravenous drug users Int J Epidemiol 1993 22 2 315 320 10.1093/ije/22.2.315 8505190

